# TGFβ Signaling in Myeloid Cells Regulates Mammary Carcinoma Cell Invasion through Fibroblast Interactions

**DOI:** 10.1371/journal.pone.0117908

**Published:** 2015-01-28

**Authors:** Aubie K. Shaw, Michael W. Pickup, Anna Chytil, Mary Aakre, Philip Owens, Harold L. Moses, Sergey V. Novitskiy

**Affiliations:** Department of Cancer Biology, Vanderbilt-Ingram Cancer Center, Vanderbilt University, Nashville, TN, United States of America; University of Kentucky College of Medicine, UNITED STATES

## Abstract

Metastasis is the most devastating aspect of cancer, however we know very little about the mechanisms of local invasion, the earliest step of metastasis. During tumor growth CD11b^+^Gr1^+^ cells, known also as MDSCs, have been shown to promote tumor progression by a wide spectrum of effects that suppress the anti-tumor immune response. In addition to immunosuppression, CD11b^+^Gr1^+^ cells promote metastasis by mechanisms that are currently unknown. CD11b^+^Gr1^+^ cells localize near fibroblasts, which remodel the ECM and leave tracks for collective cell migration of carcinoma cells. In this study we discovered that CD11b^+^Gr1^+^ cells promote invasion of mammary carcinoma cells by increasing fibroblast migration. This effect was directed by secreted factors derived from CD11b^+^Gr1^+^ cells. We have identified several CD11b^+^Gr1^+^ cell secreted proteins that activate fibroblast migration, including CXCL11, CXCL15, FGF2, IGF-I, IL1Ra, Resistin, and Shh. The combination of CXCL11 and FGF2 had the strongest effect on fibroblast migration that is associated with Akt1 and ERK1/2 phosphorylation. Analysis of subsets of CD11b^+^Gr1^+^ cells identified that CD11b^+^Ly6C^high^Ly6G^low^ cells increase fibroblast migration more than other myeloid cell populations. Additionally, tumor-derived CD11b^+^Gr1^+^ cells promote fibroblast migration more than splenic CD11b^+^Gr1^+^ cells of tumor-bearing mice. While TGFβ signaling in fibroblasts does not regulate their migration toward CD11b^+^Gr1^+^ cells, however deletion of TGFβ receptor II on CD11b^+^Gr1^+^ cells downregulates CXCL11, Shh, IGF1 and FGF2 resulting in reduced fibroblast migration. These studies show that TGFβ signaling in CD11b^+^Gr1^+^ cells promotes fibroblast directed carcinoma invasion and suggests that perivascular CD11b^+^Ly6C^high^Ly6G^low^ cells may be the stimulus for localized invasion leading to metastasis.

## Introduction

Metastasis is a key problem in cancer. Approximately 90% of patients die directly or indirectly because of the spread of cancer [1]. In breast cancer, approximately 5% of patients are diagnosed with Stage IV (SEER 1975–2008); thus 95% of patients are diagnosed with cancer that has no clinical evidence of metastasis. After treatment of their primary cancer, 11% of women will have recurrence within 5 years of treatment and 20% of women will have recurrent cancer within 10 years of treatment [2]. Treatment of these women with metastasis inhibitor drugs could prevent cancer recurrence; however, preventative therapies are limited because of lack of basic knowledge on the earliest steps of metastasis.

Intravital imaging has shown that very few cells within a tumor are motile. Motile cells localize to perivascular areas that are enriched in tumor-associated macrophages (TAM) and extracellular matrix (ECM) [3]. Stromal cells within the tumor microenvironment enhance cancer migration by secretion of chemokines and acting as leader cells for single cell or collective migration [4]. TAMs enhance breast cancer cell migration using EGF and M-CSF paracrine signaling [5,6]. Fibroblasts lead squamous cell carcinoma collective migration in tracks created by force and protease-mediated ECM remodeling [7,8]. CD11b and Gr1 cell surface markers delineate immature myeloid cells, which during tumor progression may differentiate into macrophages, dendritic cells or granulocytes. CD11b^+^Gr1^+^ double positive cells, known as myeloid derived suppressor cells (MDSCs), accumulate in pathological conditions, including infection, trauma, and tumors [9]. In tumors, MDSCs suppress T cell activity, modulate the inflammatory cytokine production of macrophages, promote angiogenesis, and enhance metastasis [9]. The mechanism of their promotion of metastasis has not been elucidated, but the cells accumulate in the invasive edges of tumors [10] and populate the lung prior to growth of lung metastatic breast cancer cells [11]. Orthotopic tumors composed of tumors cells and CD11b^+^Gr1^+^ cells increase the lung metastasis of breast cancer cells [10]. In patients, myeloid cells expressing MDSC markers are increased in cancer patients, increase with cancer stage and increase with the degree of metastasis [12].

The role of TGFβ signaling in tumorigenesis and metastatic progression is controversial. At early stages, TGFβ inhibits tumor initiation and progression by inducing cell cycle arrest and apoptosis, but at later stages of epithelial tumorigenesis it is thought to promote malignancy [13,14,15]. In our laboratory, we have shown that conditional deletion of TGFβ receptor type II (TβRII) in mammary epithelial cells resulted in shortened tumor latency and increased lung metastases [16]. In the pancreas, epithelial specific deletion of TβRII in combination with knockin of an activated Kras results in the development of much more aggressive pancreatic ductal adenocarcinomas than activated Kras alone [17]. Deletion of one allele of the type II TGFß receptor gene, *Tgfbr2*, in fibroblasts promotes metastasis in MMTV-PyMT mammary tumors and the mechanism is dependent on CXCL12 and CCL2 chemokines [18]. A number of experiments have demonstrated the importance of TGFβ signaling in immune cells, the results of which are strongly dependent on the type of cell from which TGFβ was deleted. For instance, loss of TGFβ signaling in T cells results in an autoimmune disease with early postnatal lethality [19], while selective loss of Smad4-dependent signaling in T cells leads to spontaneous gastrointestinal cancers [20]. Mice with a conditional knockout of TβRII in myeloid cells (LysM^+^) that were generated in our laboratory, showed a reduced suppressive function of CD11b^+^Gr1^+^ cells, increased antigen-presenting properties of dendritic cells and increased anti-tumorigenic properties of tumor associated macrophages (TAMs); and these changes were reflected in reduced tumor growth [21]. Subsequently, Pang at al. used the same mouse model and showed that TGFβ signaling in myeloid cells is indeed essential for tumor metastasis by regulating the production of type II cytokines, TGFβ1, arginase 1, and iNOS [22]. We recently showed that TGFβ signaling regulates expression of CD73 on mature myeloid cells which limits adenosine production and decreased metastasis in MMTV-PyMT mice associated with decrease tumor angiogenesis in parallel with increased T cell activation [23].

We hypothesized that TGFβ signaling in CD11b^+^Gr1^+^ cells would enhance the migration/invasion of cancer cells by acting on fibroblasts. We have shown that the inflammatory CD11b^+^Gr1^+^ cells promote invasion of breast carcinoma cells by directing the invasion of “leader” fibroblasts. A specific subset of CD11b^+^Gr1^+^ cells, the monocytic subtype (Ly6C^high^), secrete CXCL11, FGF2, IGF-I and Shh that promote fibroblast migration. Abrogation of TGF03B2 signaling in these myeloid cells significantly decreased fibroblast migration. This identifies a new mechanism of breast cancer local invasion and suggests new targets for metastasis prevention therapeutics.

## Materials and Methods

### Cells

Generation of Tgfbr2^fl/fl^ mice has been described previously [24]. Immortalized Tgfbr2^fspWT^ or Tgfbr2^fspKO^ fibroblasts were generated from adult mouse mammary glands as described [25]. Briefly, tumors were minced and placed in a culture flask with DMEM containing 10% FBS. After cells reached confluence, fibroblasts were differentially trypsinized from carcinoma cells using TrypLE (Life Technologies, Grand Island, NY) exposure for 30 seconds. Rapidly detached cells were re-plated to flasks. Primary fibroblasts were verified by morphology and qRT-PCR: fibroblasts are spindle shaped and express vimentin, smooth muscle actin and FSP1; and do not express E-cadherin or EpCAM. Immortalized PMTB6–2 mammary carcinoma cells were generated from MMTV-PyMT mouse mammary gland tumors as described previously [26]. Fibroblasts were maintained in DMEM containing 10% adult bovine serum and PMTB6–2 carcinoma cells were maintained in DMEM/F12 containing 5% adult bovine serum. 4T1 and LLC mouse tumor cell lines were obtained from American Type Culture Collection (Manassas, VA, USA) and maintained following the manufacturer’s protocols.

### Animals and orthotopic grafts

Orthotopic grafts were prepared by suspending 500,000 4T1 cells in 25 mkl of neutralized rat tail collagen. Grafts were placed in inguinal fatpads of female Balb/c mice. Spleens and tumors were collected 3–5 weeks after tumor palpation or graft implantation. LLC cells (5×10^5^ cells) were injected s.c. into the right flank of c57bl/6 mice. TGFβRII^MyeKO^ and TGFβRII^MyeWT^ mice, on a C57BL6 background, were established and maintained as described [21]. Naïve Balb/c and c57bl/6 mice were ordered from The Jackson Laboratory (Bar Harbor, ME, USA).

All mice were housed in the Department of Animal Care at Vanderbilt University Medical Center following the Association for the Assessment and Accreditation of Laboratory Animal Care and Institutional Animal Care and Use Committee guidelines. The studies were approved by IACUC at Vanderbilt University Medical Center protocol #M/07/331 regulating animal welfare to ameliorate any unnecessary suffering. Animals were sacrificed using CO2 asphyxiation.

### Magnetic Separation of CD11b^+^Gr1^+^ cells

Splenocytes were isolated by passage of dissected spleen through 70 um nylon cell strainers. Pellets were incubated in ACK buffer (0.15M NH_4_Cl, 10mM KHCO_3_, 0.1mM EDTA) for 5 minutes to lyse erythrocytes. Gr1^+^ splenocytes were collected using MACS magnetic microbead separation (Miltenyi Biotech, Auburn, CA). Cleared splenocytes were incubated with biotinylated anti-Gr1 antibody (BD Biosciences, San Jose, CA 553125) and Streptavidin microbeads (Miltenyi Biotec) according to manufacturer instructions. CD11b^+^Gr1^+^ cells were collected from MACS LS Columns and the indicated cell numbers were cultured for 16–18 hours in RPMI 1640 containing 55 um 2-mercaptoethanol and 10% FBS. Conditioned medium (CM) was generated by culturing 2.5 million cells for 16–18 hour and centrifuging culture medium at 1500 g and passing supernatant through a 0.22 um filter.

### Migration assay

6.5 mm Transwell polycarbonate inserts with 8 um pores (Corning, Lowell, MA, cat #3422) were coated with 1 mg/ml fibronectin containing 0.1% gelatin. Cells, CM or medium was placed in the lower chamber and the insert was replaced. Fibroblasts were cultured in serum-free DMEM for 12 hours. 50,000 fibroblasts were placed on the Transwell insert and cells were allowed to migrate for 5 hours. Cells were fixed in 10% neutral buffered formalin and stained with Mayer’s hematoxylin solution. Non-migrated cells were removed from the top of the Transwell filter using a cotton swab. Filters were cut out and mounted on microscope slides. Migrated cells were counted by imaging 10 random fields of view and cells were counted using ImageJ 1.43u (NIH).

### Fluorescent invasion assay

6.5 mm Transwell inserts were coated with 1 mg/ml Growth Factor Reduced Matrigel (BD Biosciences). Cells, CM or medium was placed in the lower chamber and the insert was replaced. Cells were cultured in serum-free DMEM for 12 hours. Fibroblasts were labeled with 1,1′-dioctadecyl-6,6′-di(4-sulfophenyl)-3,3,3′,3′-tetramethylindocarbocyanine (Life Technologies, Grand Island, NY) according to manufacturer instructions. PMTB6–2 carcinoma cells were labeled with 1,1′-dioctadecyl-6,6′-di(4-sulfophenyl)-3,3,3′,3′-tetramethylindocarbocyanine (Life Technologies) according to manufacturer instructions. 50,000 fibroblasts and/or 50,000 carcinoma cells were placed on the Transwell insert and cells were allowed to migrate for 16 hours. Cells were fixed in 1% paraformaldehyde and counterstained with DAPI. Non-invaded cells were removed from the top of the filter using a cotton swab. Filters were cut out and mounted on microscope slides. Invaded cells were counted by imaging 10 random fields of view using Texas Red and FITC fluorescent microscope filters. Cells of each fluorescent color were overlaid with DAPI and counted manually. Migration assays with purified recombinant proteins were treated in RPMI 1640 containing 0.1% FBS with the midpoint of the ED_50_ of the listed dose according to manufacturer specifications (R&D Systems, Minneapolis, MN). ED_50_ doses: 10ng/ml CXCL11, 50ng/ml CXCL15, 1ng/ml FGF2, 1ng/ml IGF-I, 10ng/ml IGF-II, 60ng/ml IL-1ra, 2 μg/ml Resistin, 0.5 μg/ml Shh, 0.5 μg/ml VEGF-D, 20ng/ml VEGFR1.

### Western immunoblots and quantitation

10 cm dishes were coated with 1mg/ml fibronectin containing 0.1% gelatin and 7 million cells in DMEM containing 10% adult bovine serum. After 24 hours, medium was replaced with serum-free DMEM. Serum-starved cells were then treated with RPMI 1640 containing 55 um 2-mercaptoethanol and 10% FBS (untreated) or CD11b^+^Gr1^+^ cells CM. Lysates were collected in TNE buffer (10mM Tris, 0.2M NaCl, 1mM EDTA, pH 7.4) containing protease inhibitor cocktail and phosphatase inhibitor cocktail 3 (Sigma-Aldrich, St. Louis, MO). Protein was quantitated using Bradford assay and 30 ug of total protein was separated by SDS-PAGE. Protein was transferred to nitrocellulose membranes and blocked with 5% BSA or 5% nonfat dry milk diluted in TBST. Proteins were immunoblotted using the following antibodies: Erk1/2 (Santa Cruz, Santa Cruz, CA sc-94), p-Erk1/2 (Santa Cruz sc-7383), p38 (Cell Signaling, Boston, MA 9212), p-p38 (Cell Signaling 9211), c-Src (Santa Cruz sc-18), p-cSrc (Life Technologies 44–660G), Akt1 (Cell Signaling 2967), p-Akt1 (Cell Signaling 9271), JNK (Cell Signaling 9258), p-JNK (Cell Signaling9255), PI3K p85 (Cell Signaling 4257), p-PI3K p85 (Cell Signaling 4228), FAK (Cell Signaling 3285), p-FAK Y397 (Cell Signaling 3283) and secondaries ant-rabbit-HRP or anti-mouse-HRP (Jackson ImmunoResearch, West Grove, PA). Proteins were detected by enhanced chemiluminescence (Pierce, Rockford, IL) and captured on photographic film. Films were scanned on HP flatbed scanner and densitometry was completed using ImageJ.

### Antibody array (CM)

Untreated medium consists of RPMI 1640 containing 55 ᷭm 2-mercaptoethanol and 10% FBS. Three million freshly isolated CD11b^+^Gr1^+^ cells were cultured for 16 hours in the same medium. Serum-starved immortalized Tgfbr2^fspWT^ fibroblasts were cultured in the same medium for 16 hours. Serum-starved immortalized Tgfbr2^fspWT^ fibroblasts were cultured in 16 hour CD11b^+^Gr1^+^ cells CM for 16 hours. Conditioned medium was collected and centrifuged at 1500g for 5 minutes. Supernatants were passed through 0.22 um filters. Samples were exposed to RayBiotech Mouse Cytokine Antibody Array C Series 2000 (Norcross, GA) according to manufacturer instructions. Films were scanned on HP flatbed scanner and densitometry was completed on ImageJ. All protein optical densities were normalized to positive controls on each membrane.

### qPCR (primers)

RNA was isolated using RNeasy mini kit (Qiagen, Valencia, CA) with on-column DNase digestion. Total RNA (1 ug) was reverse transcribed to generate cDNA using M-MLV reverse transcriptase (Life Technologies). Relative mRNA quantity was determined by real-time RT-PCR using iCycler instrumentation and software (BioRad, Hercules, CA). Primer sequences are available by request.

### Flow cytometry

Splenocytes were prepared as described above. 4T1 tumor explants were finely chopped and digested in 1mg/ml collagenase I (Sigma C0130) and 1mg/ml Dispase II (Roche 11629200) for 45 minutes at 37C. 10 uU/ml DNase (Calbiochem 260913) was added and incubated for 5 minutes. Tumor cells were gently pushed through a 70 um cell strainer and gently washed several times in cold PBS. Cells were blocked with Fc block (BD Biosciences). Cells were labeled with antibodies specific for: CD45-APC (Biolegend, San Diego, CA 103111), CD45-PE/Cy7 (Biolegend 103113), CD11b-FITC (BD Biosciences 553310), CD11b-APC (Biolegend 101211), Gr1-PE (Biolegend 108407), Ly6C-FITC (BD Biosciences 553104), Ly6G-PE (Biolegend 127607), CD3-FITC (Biolegend 100203), CD19-PE (Biolegend 115507) and counterstained with DAPI. CD11b^+^Gr1^+^ cells, Ly6C, Ly6G, monocytes, B cells and T cells were collected by FACS using BD FACSAria III instrumentation (BD Biosciences). Flow cytometry experiments were performed in the VUMC Flow Cytometry Shared Resource. The VUMC Flow Cytometry Shared Resource is supported by the Vanderbilt-Ingram Cancer Center (P30 CA68485) and the Vanderbilt Digestive Disease Research Center (DK058404). CM was generated as described above using 1 million cells/ml.

### Statistical analysis

Data were analyzed using the GraphPad Prism 5.02 software (GraphPad Software Inc., San Diego, CA) and presented as mean ± SEM. Comparisons between treatment groups and control untreated groups were performed using one-way ANOVA followed by Dunnett’s posttests. Comparisons between two groups were performed using two-tailed unpaired *t* tests. A *P* value < .05 was considered significant.

## Results

### CD11b^+^Gr1^+^ cells secretions promote fibroblast migration

**Fig 1:**
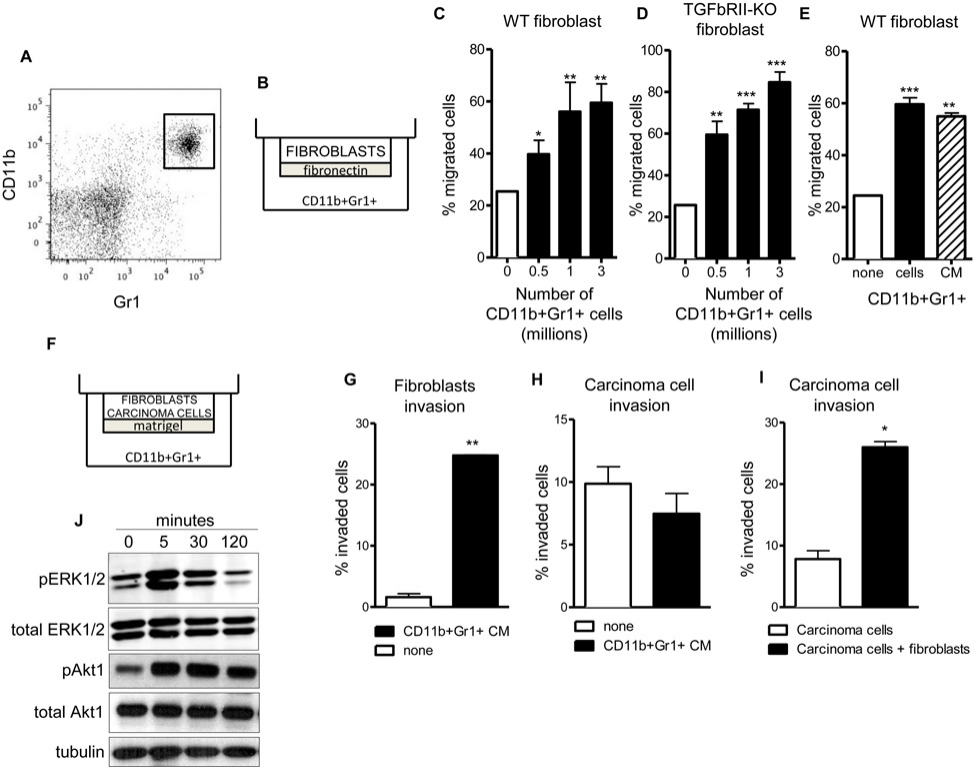
CD11b^+^Gr1^+^ cells increase migration of fibroblasts and invasion of co-cultured carcinoma cells. (**A**) CD11b^+^Gr1^+^ cells were isolated from spleen of tumor-bearing mice by magnetic separation and cultured for 16–18 hours. (**B**) Fibronectin-coated 8 uM pore Transwells were placed on top of cultured CD11b^+^Gr1^+^ cells and 5x10^4^ mammary fibroblasts were allowed to migrate for 5 hours. (**C**) CD11b^+^Gr1^+^ cells dose-dependently increase fibroblast migration. (**D**) Fibroblasts with deletion of TGFβRII have a further increase in migration in response to CD11b^+^Gr1^+^ cells. (**E**) Conditioned medium (CM) collected from CD11b^+^Gr1^+^ cells increases fibroblast migration to the same extent as live CD11b^+^Gr1^+^ cells. * – p<0.05, ** – p<0.01, *** – p<0.001 compared to no CD11b^+^Gr1^+^ cells. (**F**) Matrigel-coated 8 uM pore Transwells were placed on top of CD11b^+^Gr1^+^ CM and mammary carcinoma cells, fibroblasts or both cell types were allowed to invade for 18 hours. (**G**) CD11b^+^Gr1^+^ cells increase fibroblasts invasion. (**H**) CD11b^+^Gr1^+^ cells do not increase the invasion of carcinoma cells. (**I**) CD11b^+^Gr1^+^ cells increase carcinoma cell invasion when fibroblasts are co-cultured. * – p<0.05, ** – p<0.01, compared to open bars. (**J**) Western immunoblot of fibroblast Erk1/2 and Akt phosphorylation shows activation after 5 minutes of treatment with CM of CD11b^+^Gr1^+^ cells.

We isolated CD11b^+^Gr1^+^ cells from spleens of mice bearing orthotopic 4T1 mammary gland tumors using Gr1 antibody targeted magnetic separation. CD11b^+^Gr1^+^ cells can be isolated using a single antibody since all Gr1^+^ cells in the spleen are also CD11b^+^ (Fig. 1A). Freshly isolated CD11b^+^Gr1^+^ cells were used as the attractant for fibroblasts migrating through a fibronectin matrix (Fig. 1B). CD11b^+^Gr1^+^ cells promote migration of immortalized mouse mammary gland fibroblasts in direct response the number of CD11b^+^Gr1^+^ cells (Fig. 1C). Since CD11b^+^Gr1^+^ cells secrete TGFβ ligand [10], we wanted to determine if TGFβ responsiveness in fibroblasts is required for this induced migration. Immortalized fibroblasts that lack TGFβRII, and do not respond to TGFβ ligand, were placed in a migration chamber with different numbers of live CD11b^+^Gr1^+^ cells. CD11b^+^Gr1^+^ cells increase migration of TGFβRII-deficient fibroblasts, indicating that TGFβ signaling is not required for CD11b^+^Gr1^+^ cells stimulation of fibroblast migration (Fig. 1D). To determine if CD11b^+^Gr1^+^ cells secrete products that promote fibroblast migration, we prepared conditioned medium (CM) from CD11b^+^Gr1^+^ cells cultured for 16–18 hours. Fibroblasts exposed to live CD11b^+^Gr1^+^ cells or CD11b^+^Gr1^+^ cells CM increased migration equally, indicating that secretions from CD11b^+^Gr1^+^ cells drive fibroblast migration (Fig. 1E). Immortalized fibroblasts may have an altered migration response, so we examined primary fibroblast migration and found the same response (data not shown).

Our migration assay was converted into an invasion assay by providing a thick layer of Matrigel basement membrane for cells to invade through (Fig. 1F). Fibroblasts and carcinoma cells were labeled by different fluorochrome dyes (M&M section). Immortalized mouse mammary fibroblasts invade through Matrigel when attracted by CD11b^+^Gr1^+^ cells CM (Fig. 1G). Interestingly, CM from CD11b^+^Gr1^+^ cells does not stimulate invasion of mouse mammary carcinoma cells (Fig. 1H) unless they are co-cultured with fibroblasts (Fig. 1I).

To understand the mechanism of communication between CD11b^+^Gr1^+^ cells and fibroblasts, we examined induced signal transduction pathways in fibroblasts treated with CD11b^+^Gr1^+^ cells CM. We examined phosphorylation of p38 MAPK, c-Src, JNK, PI3K p85, FAK and found no alterations (data not shown). We did find increased phosphorylation of ERK1/2 and Akt1. ERK1/2 was rapidly phosphorylated after five minutes of CD11b^+^Gr1^+^ cells CM treatment and returned to basal levels after 2 hours (Fig. 1J). Akt1 was phosphorylated at 5 minutes of CD11b^+^Gr1^+^ cells CM treatment and continued to rise over time (Fig. 1J).

### Identification of proteins secreted by CD11b^+^Gr1^+^ cells

**Fig 2:**
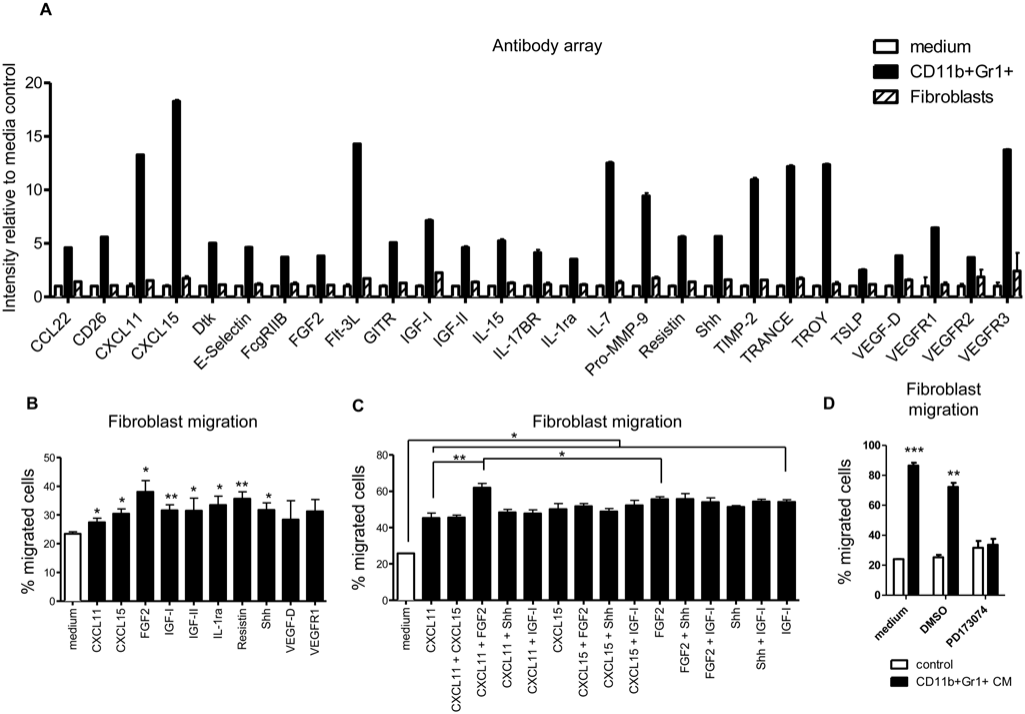
Cytokines and chemokines secreted by CD11b^+^Gr1^+^ cells increase fibroblast migration. (**A**) Antibody array analysis of proteins secreted by CD11b^+^Gr1^+^ cells compared to fibroblasts. Although 144 proteins were assayed, only proteins predominantly expressed in CD11b^+^Gr1^+^ cells are shown. (**B**) Purified cytokine treatment of fibroblasts increases migration through Transwell chambers. (**C**) Combinations of several cytokines and chemokines increase fibroblast migration beyond treatment with a single cytokine/chemokine. Concentrations are same as on (B), * – p<0.05, ** – p<0.01, *** – p<0.001 compared to untreated; #: p<0.01 compared to all other treatments. (**D**) Inhibition of FGFR1 Kinase by PD173074 (5 nM) prevents fibroblast migration towards conditioned medium from CD11b^+^Gr1^+^ cells. ** – p<0.01, *** – p<0.001 relative to control medium.

To identify the CD11b^+^Gr1^+^ cells cytokines that mediate fibroblast invasion, we analyzed conditioned medium from CD11b^+^Gr1^+^ cells using an antibody array of 144 secreted proteins. We compared cytokines from CD11b^+^Gr1^+^ cells and fibroblasts and identified 27 cytokines from CD11b^+^Gr1^+^ cells that are not secreted by fibroblasts (Fig. 2A). These include proteins regulating cell growth, immune cell recruitment, and immunogenic function. Comparison of fibroblasts and CD11b^+^Gr1^+^ cells showed that 7 proteins are predominantly expressed in CD11b^+^Gr1^+^ cells. These proteins include CXCL11, CXCL15, IGF-I, IL1ra, Resistin, VEGF-D and VEGFR1.

To identify specific factors responsible for CD11b^+^Gr1^+^ cells enhancement of fibroblast migration, we treated fibroblasts with purified proteins that were identified above. We tested 10 recombinant proteins and found that 8 increased fibroblast migration (Fig. 2B). CXCL11, CXCL15, FGF2, IGF-I and Shh further increased fibroblast migration in a dose dependent manner (data not shown). Combining CXCL11 and FGF2 increased fibroblast migration above CXCL11 or FGF2 alone (Fig. 2C). Because FGF2 had a more promising effect in stimulation of fibroblast migration, alone or in combination with CXCL11, we used an FGFR3 inhibitor (PD173074) to determine if pharmaceutical inhibition can decrease migration of fibroblasts toward CD11b^+^Gr1^+^ cells. We found that the FGFR3 inhibitor can completely abolish the stimulated effect of CD11b^+^Gr1^+^ cells on fibroblast migration (Fig. 2D).

### CD11b^+^Ly6C^hgh^Ly6G^low^ cells are the primary mediator of fibroblast migration and it is dependent on TGFβ signaling

**Fig 3:**
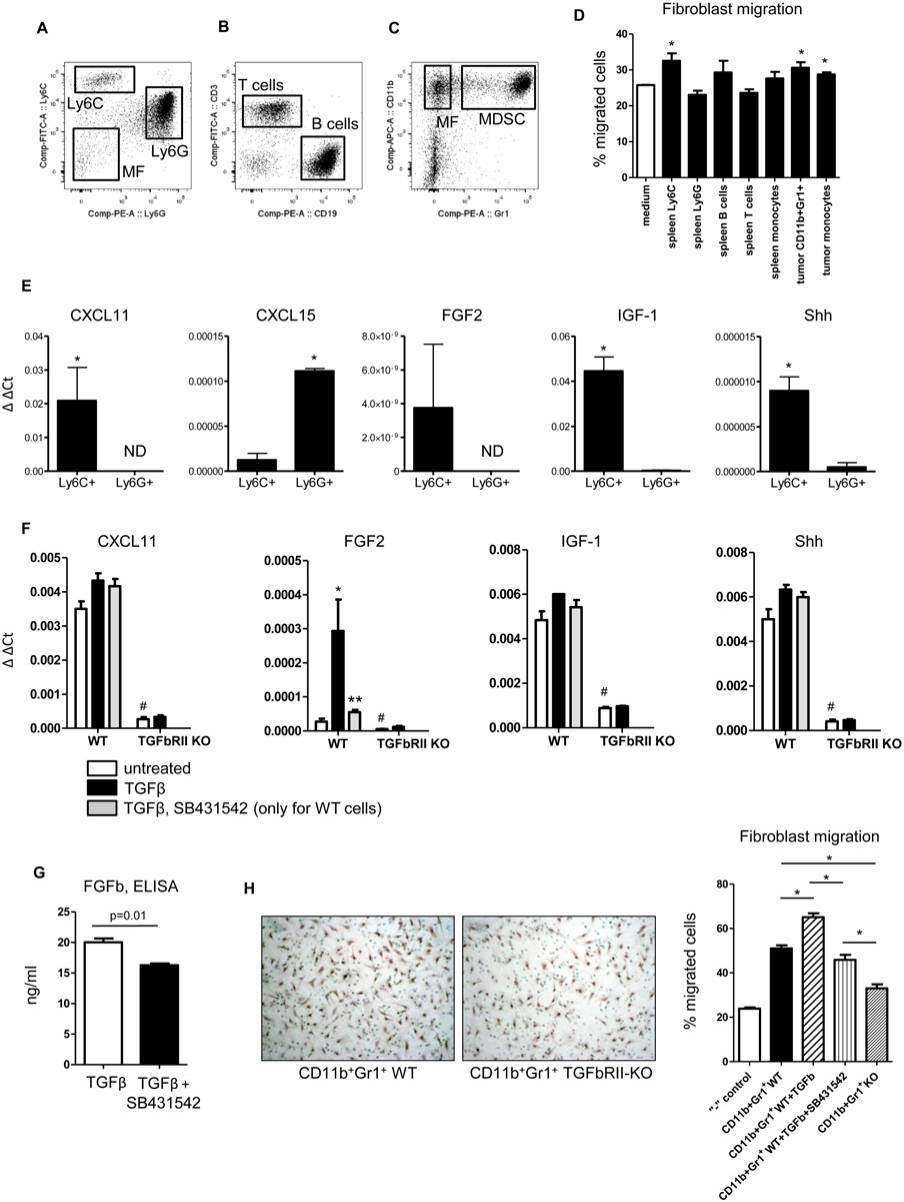
TGFβRII signaling in Ly6C^high^Ly6G^low^ cells regulates the secretion of chemokines and results in increased fibroblast migration. (**A**) FACS plot showing collection of Ly6C^+^, Ly6G^+^ and monocytes from spleen of 4T1 tumor-bearing mice. The plot was gated for CD45^+^ and CD11b^+^ cells. (**B**) FACS plot showing collection of T cells (CD3^+^) and B cells (CD19^+^) from spleen of 4T1 tumor-bearing mice. The plot was gated for CD45^+^ cells. (**C**) FACS plot showing collection of CD11b^+^Gr1^+^ cells and macrophages (CD11b^+^Gr1^+^) from 4T1 tumor tissue. The plot was gated for CD45^+^ cells. (**D**) Fibroblast migration to conditioned medium (from 1x10^6^ cells) prepared from subsets of splenocytes or myeloid cells from spleen and tumor tissue. Ly6C^+^ cells from spleen, tumor-derived CD11b^+^Gr1^+^ cells and tumor macrophages induce the most fibroblast migration. * – p<0.05 compared to control. (**E**) qRT-PCR analysis of cytokines/chemokines in splenic Ly6G (CD11b^+^Ly6G^high^Ly6C^low^) and Ly6C (CD11b^+^Ly6C^high^Ly6G^low^) cells. (**F**) qRT-PCR analysis of cytokines/chemokines in splenic CD11b^+^Gr1^+^ cells stimulated by TGFβ1 (1 ng/ml) for 18hr. with and without SB431542 (10 uM) * – p<0.05 compared with untreated cells, ** – p<0.05 compared with TGFβ treated cells, # – p<0.05 compared with WT cells. (**G**) CD11b^+^Gr1^+^ cells (4x10^6^) isolated from spleen of LLC tumor bearing mice were incubated 18hr in presence of TGFβ (1ng/ml) and TGFβ and SB431542 (10uM). Level of FGFb was measured by ELISA (R&D System, Minneapolis, MN). (**H**) CD11b^+^Gr1^+^ cells (3x10^6^) isolated from spleen of LLC tumor bearing mice with deleted TGFβRII decrease migration of fibroblasts compared with CD11b^+^Gr1^+^ cells with intact TGFβ signaling. “-” control—negative control, DMEM without serum, * – p<0.01. TGFβ1 (1 ng/ml), SB431542 (10 uM).

CD11b^+^Gr1^+^ cells are composed of different subsets of myeloid cells [27]. We collected CD11b^+^, Ly6G^low^Ly6C^high^ cells (Ly6C, M-MDSC), CD11b^+^Ly6G^high^Ly6C^low^ cells CD11b^+^Gr1^+^ cells (Ly6G, G-MDSC), and CD11b^+^Ly6G^-^Ly6C^-^ (macrophages) from the spleen of 4T1 tumor-bearing mice (Fig. 3A). We also collected B cells (CD19^+^) and T cells (CD3^+^) from the spleens of the same mice (Fig. 3B). From tumor tissue (4T1), we collected CD11b^+^Gr1^+^ cells and CD11b^+^Gr1- cells (Fig. 3C). The cells were cultured for 16–18 hours and conditioned medium was collected. We examined fibroblast migration to immune cell CM and found that spleen Ly6C, tumor CD11b^+^Gr1^+^ cells and tumor macrophages increased fibroblast migration (Fig. 3D). Confirming our chemokine profile, analysis of gene expression in Ly6C and Ly6G cells showed that CD11b^+^Ly6C^high^Ly6G^low^ cells (Ly6C) have increased expression of CXCL11, FGF2, IGF-I and Shh relative to CD11b^+^Ly6G^low^Ly6G^high^ cells (Ly6G) (Fig. 3E).

We reported previously that a significant number of cytokines/chemokines expression by myeloid cells are regulated by TGFβ signaling [21] as well as adenosine production [23]. Further examination of chemokine expression revealed an enrichment of CXCL11, FGF2, IGF-1 and Shh in CD11b^+^Gr1^+^ cells isolated from spleen of LLC tumor bearing transgenic mice that lack TGFβRII only on myeloid cells (LysM^+^). Basal levels of CXCL11, IGF-1 and Shh expression was dramatically decreased in CD11b^+^Gr1^+^/TβRII-KO cells vs. CD11b^+^Gr1^+^ WT cells and not changed by TGFβ stimulation (Fig. 3F). Basal expression of FGF2 in KO cells was decreased 3 fold but highly upregulated by TGFβ on WT cells compared to other cytokines. Adding the TGFβ signaling inhibitor, SB431542, to WT cells inhibits only FGF2 expression in CD11b^+^Gr1^+^ cells. By ELISA we found similar effect of SB431542 on TGFβ stimulated secretion of FGFb by CD11b^+^Gr1^+^ cells (Fig. 3G). In a migration assay we found that TGFβ can stimulate the ability of CD11b^+^Gr1^+^ cells to increase migration of fibroblasts and adding SB431542 decreases this effect. However, KO cells still have lower ability to stimulate fibroblast migration vs. using TGFβ signaling inhibitor (Fig. 3H) probably due to an incompleted inhibition of canonical and noncanonical TGFβ signaling pathways.

## Discussion

The significant finding in this study is that TGFβ signaling in monocytic MDSCs (CD11b^+^Ly6C^high^Ly6G^low^) stimulates the migration of fibroblasts within tumors and that this increases the invasion of carcinoma cells. Our data integrate with two previously observed findings—first; the increased number of MDSC correlates with metastasis in human cancer patients [28] and second; myeloid cells from patients with advanced-stage cancer have increased TGFβRII expression [22]. We discovered that FGF2 together with CXCL11, IGF-I and Shh secreted by CD11b^+^Gr1^+^ cells mediates fibroblast migration. This points to a novel role for CD11b^+^Gr1^+^ cells in metastasis by directing local invasion.

Metastasis requires several steps. 1) Angiogenesis – blood vessels grow into a tumor from nearby vessels; 2) The next step, which we know very little about is the invasion of cancer cells through their microenvironment toward newly developed blood vessels; 3) Intravasation – cancer cells become able to traverse the vascular wall to enter a blood vessel; 4) Once cancer cells enter the vascular compartment, they are continually assaulted with immunosuppressive cells, lack of adhesion and sheer force and some cells activate survival mechanisms; 5) Extravasation – surviving cancer cells can become arrested in micro-capillaries and exit the blood vessel; 6) Extravasated cancer cells may find a hospitable microenvironment in their new locale and begin to grow to form a secondary micro-metastatic tumor. 7) Finally, the secondary tumor stimulates angiogenesis to provide nutrients and dispose of waste to form a clinically detectable metastatic tumor [29]. The current state of anti-metastasis therapy is to treat with angiogenesis inhibitors. Vascular Endothelial Growth Factor (VEGF) is one of many proteins that stimulate angiogenesis. A monoclonal antibody that blocks the effects of VEGF, Bevacizumab/Avastin, is currently being used as therapy in several types of cancer [30]. However, the FDA recently withdrew Avastin from breast cancer treatment as it was shown that the drug was ineffective and unsafe [31]. Aside from anti-angiogenesis therapies, we have no other options to prevent breast cancer metastasis in patients with localized breast cancer. Blockade of local invasion could be used as a secondary mechanism to prevent metastasis.

CD11b^+^Gr1^+^ cells secrete many cytokines, chemokines, growth factors and proteases. We have identified a set of proteins that activate migration and chemotaxis of fibroblasts. We suggest that FGF2, especially in combination with CXCL11, has a primary effect on fibroblast migration. FGF2 is expressed in luminal and myoepithelial cells of the normal mammary gland, but is lost in breast cancer [32]. Re-expression of FGF2 in motile MDA MB 231 breast cancer cells results in activation of focal adhesion and loss of motility [33]. FGF2 stimulates the proliferation and migration of fibroblasts during wound healing and activates angiogenesis [34]. Since cell adhesion is maintained in collectively migrating cancer cells [35], FGF2 secreted by CD11b^+^Gr1^+^ cells may induce adhesion of cancer cells and migration of fibroblasts to enhance collective migration of breast cancer cells.

CXCL11, also known as Interferon-inducible T-cell alpha chemoattractant, acts on target cells by activation of CXCR3 and has the highest affinity for the receptor compared with its other ligands [36]. CXCR3 has been associated with invasion of several different types of cancer, including breast cancer and is expressed in all human breast cancer cell lines. Antagonism of CXCR3 with the small molecule AMG487 in mice bearing syngeneic subcutaneous breast tumors resulted in decreased lung metastasis, but did not affect growth of the primary tumor [37]. CD11b^+^Gr1^+^ secretion of CXCL11 may mediate effects on fibroblasts as well as tumor cells.

IGF-1 is over-expressed in breast cancer [38]. IGF-1 mediates cancer cell migration, invasion and angiogenesis and mediates its intracellular signal by activation of IGF1R-PI3K-Akt signaling pathway [39]. In breast cancers treated with EGFR inhibitors, IGF1R can replace EGFR in heterodimers to confer resistance [40]. This suggests that IGF1R may play a role in the EGF-MCSF cancer cell-TAM paracrine invasion pathway. Inhibitors of IGF1R activity are being strongly pursued for anti-cancer therapeutics [41,42].

Breast cancer local invasion is moderated by the tumor microenvironment. This is the first observation of paracrine communication between myeloid cells (CD11b^+^Gr1^+^) and fibroblasts in mediating mammary carcinoma cell invasion by TGFβ regulation of the secretion of pro-invasive cytokines/chemokines. Future studies should examine interactions between cells in the tumor microenvironment in mediating tumor progression with a focus on myeloid cell-specific TGFβ signaling.
